# Supplementary material for: Monoclonal Antibodies Can Aid in the Culture-Based Detection and Differentiation of Mucorales Fungi—The Flesh-Eating Pathogens Apophysomyces and Saksenaea as an Exemplar
Source: Antibodies (Basel). 2025 Oct 7;14(4):85. doi: 10.3390/antib14040085 (PMC12550967; doi:10.3390/antib14040085)
Supplement: Supplementary file 1 [file antibodies-14-00085-s001.zip › antibodies-3895819-supplementary.pdf]

antibodies

Article

Monoclonal antibodies can aid in the culture-based detection and differentiation of *Mucorales* fungi—the flesh-eating pathogens *Apophysomyces* and *Saksenaea* as an exemplar

Christopher R. Thornton and Genna E. Davies

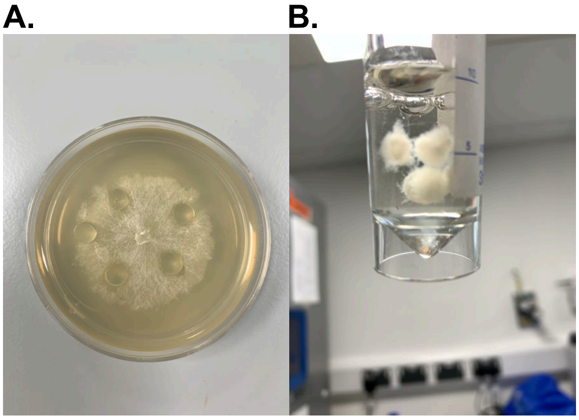

Supplementary Figure S1. (**A**) Culture of *A. variabilis* CBS658.93 after 24 h growth on MEA at 37 °C. Note the removal of the five mycelial discs using a cork borer prior to antigen extraction for TG11-LFD swab tests. (**B**) Colonies of *A. variabilis* after 24 h incubation of mycelial discs in YNB+G medium at 37 °C with shaking (60 RPM).

 https://doi.org/10.3390/antib14040085
